# How Official Social Media Affected the Infodemic among Adults during the First Wave of COVID-19 in China

**DOI:** 10.3390/ijerph19116751

**Published:** 2022-05-31

**Authors:** Huan Liu, Qiang Chen, Richard Evans

**Affiliations:** 1 School of Journalism and New Media, Xi’an Jiaotong University, Xi’an 710049, China; hustcq@163.com; 2 Faculty of Computer Science, Dalhousie University, Halifax, NS B3H 4R2, Canada; R.Evans@dal.ca

**Keywords:** COVID-19 infodemic, official social media, information cascades, social support, public health emergency

## Abstract

The COVID-19 pandemic has demonstrated that social media can impact society both positively (e.g., keeping citizens connected and informed) and negatively (e.g., the deliberate spreading of misinformation). This study aims to examine the underlying mechanisms of the relationship between official social media accounts and the infodemic, experienced during the first wave of COVID-19 in China. A theoretical model is proposed to examine how official social media accounts affected the infodemic during this period. In total, 1398 questionnaire responses were collected via WeChat and Tencent QQ, two leading Chinese social media platforms. Data analysis was conducted using Partial Lease Squares Structural Equation Modeling (PLS-SEM), moderation effect analysis, and mediation effect analysis. Results indicate that the Information Quality (IQ) of Official social media accounts (β = −0.294, *p* < 0.001) has a significant negative effect on the infodemic. Mediation effect analysis revealed that both social support (β = −0.333, 95% Boot CI (−0.388, −0.280)) and information cascades (β = −0.189, 95% Boot CI (−0.227, −0.151)) mediate the relationship between IQ and the infodemic. Moderation effect analysis shows that private social media usage (F = 85.637, *p* < 0.001) positively moderates the relationship between IQ and the infodemic, while health literacy has a small negative moderation effect on the relationship between IQ and the infodemic. Our findings show that, in the context of Chinese media, official social media accounts act as a major source of information for influencing the infodemic through increasing social support and reducing information cascades for citizens.

## 1. Introduction

The COVID-19 pandemic has proven how social media can quickly create a parallel infodemic, impacting the health and wellbeing of global citizens and posing a challenge for the delivery of public health services, worldwide [1]. The World Health Organization (WHO) defines the term ‘infodemic’ as ‘an overabundance of information’, some of which is accurate and some not, which makes it difficult for citizens to identify trustworthy sources of information and reliable guidance [2]. At the start of the pandemic, citizens’ consumption of news increased by 62% [3], with many being exposed to mass amounts of misinformation and fake news as they searched for information relating to COVID-19 [4,5]. An infodemic typically includes the dissemination of unclear and unreliable messages, rumors, and fake news, which affects the penetration of public health communication and causes mass anxiety and social panic, ultimately impeding effective crisis management [6,7,8]. In China, in the early stage of the pandemic, false information spread rapidly on social media [9], causing serious difficulties in managing the disease [10]. Combating COVID-19 requires the combined efforts of multiple stakeholders who disseminate accurate and authoritative information through different media channels in a timely manner [11,12]. For example, governments and public health agencies should provide up-to-date reliable information on COVID-19 and emotional support to citizens in order to reduce public anxiety and uncertainty [13]. There is a societal need for accurate information to be corroborated quickly to prevent the spread of misinformation resulting in mass panic [14]. Official social media accounts, such as those managed by governments, serve as an ideal medium for facilitating communication between official sources and citizens during public health crises [15], but their strict control invalidating and disseminating information may inhibit fast and effective dissemination and lead to public distrust towards such organizations [16].

Many scholars have noted the widespread adoption of the term ‘infodemic’ by the research community [17]. However, the term still requires some clarification, especially in terms of how it is measured, which is still not fully understood. To address this shortcoming, this study provides a measurement for the current and future infodemic. Previous infodemic studies have focused predominantly on how to control them, such as minimizing the spread of fake news, misinformation, and rumors, as well as controlling their impact on citizens’ psychological health [18,19]. However, a critical question remains: How do official social media accounts affect the infodemic? The aim of this study, therefore, is multifold. First, we aim to create a conceptual framework and provide practical implications for reducing the severity of an infodemic and, second, we aim to explore the possible relationship between official social media accounts and the infodemic, in the context of public health crises. By doing so, this study contributes to both the processing of health information on official social media accounts and the understanding of how to respond to an infodemic.

## 2. Literature Review and Hypotheses

### 2.1. Theoretical Basis

The Social-Mediated Crisis Communication (SMCC) model is widely used to study how and why citizens communicate about crises, especially in terms of how different sources and forms of initial crisis information are exposed and affect follow-up crisis communication. Similarly, it describes the relationships between organizations, citizens, social media, and traditional media, during and after crises have occurred [20]. Some researchers have used the SMCC model to explore how citizens cope with risk information disseminated by governments, such as their information processing behaviors, changes in emotions, and protective behaviors [21]. The SMCC model focuses on the format and sources of information, and social media effectiveness to improve social resilience. Further, it suggests that citizens use social media to meet their untapped social needs, such as to vent, socialize with friends and family, seek information, and to obtain emotional support [22]. The emotional support provided to citizens through different media sources can directly affect their feelings and their responses [23], and one important factor of socialization is communication through social media [24].

The SMCC model categorizes information sources into either official (i.e., from public organizations, such as governments, which share crisis information with citizens) or a third party (i.e., members of the public or groups of citizens that share unverified crisis information with other citizens) [25]. Information posted by official sources that has been verified is key to establishing credibility and trust among citizens [26], but the COVID-19 infodemic resulted in the frequent sharing of misleading information and false claims, such as the sharing of pseudo-scientific therapies, and discussions about the origin and spread of the disease [27]; these activities can undermine public trust in governments. A previous study in India found that focusing on Twitter sentiment was an important crisis management strategy [28]. Therefore, to reduce the harm caused by public health crises, government agencies and public health organizations can use social media to help deal with the dissemination of crisis information [29].

Extant research shows that government social media accounts are an important information source for promoting citizen engagement during COVID-19 [30]. We posit, therefore, that official social media accounts are a key facilitator in successfully communicating with citizens and act as an important information source and provider of emotional support. During the first wave of the COVID-19 pandemic in China, citizens knew very little about the disease, causing mass panic and anxiety. If an information source is unofficial with low information quality, it can affect the emotions of citizens and lead to an infodemic, which is shown as important variables (e.g., official social media, social support, and infodemic) in the theoretical model. However, the SMCC model does not mention the variable of information cascades. When citizens know little about a crisis, information cascades can easily occur. During the initial stage of the pandemic, citizens knew very little about COVID-19, so we have, therefore, introduced the variable, information cascades, into our theoretical model based on the SMCC model.

### 2.2. Social Media in Public Health Crises (Official and Private) and the Infodemic

During the COVID-19 pandemic, governments imposed frequent lockdowns with the aim of controlling the spread of the COVID-19 disease. During these times, citizens used social media more frequently than usual [31,32], becoming compulsive and often demonstrating an addictive behavior [33]. The information posted on social media acted as a double-edged sword. First, verified information relieved citizens’ panic and anxiety and motivated them in the fight against COVID-19 [34]. Secondly, however, as the amount of information available grew, citizens became unsure about whether the information they were viewing was, in fact, true [35].

Throughout the lockdowns, social media and the Internet acted as a main source of information for citizens [36,37], with social media use rapidly increasing during the crisis [38]. Communication during the pandemic was characterized by knowledge communities, organized into hierarchies of subgroups with clear geopolitical and ideological characteristics [39]. Citizens used social media to obtain health-related information, such as to learn about necessary control measures, disseminate the latest information about the pandemic, and listen to critical announcements [40]. However, content posted to social media was not always censored like the state-controlled media [41]; this ultimately affected the spread of anxiety and fear among citizens [42].

As the pandemic evolved, information related to COVID-19 received far greater attention than non-COVID-19 information on commercial social media platforms [43]. The infodemic started to portray the characteristics of repeated fluctuations [44,45], resulting in vast amounts of misinformation and fake news, demonstrating different types of reconfigured and fabricated content and dubious ideas [46]. The emergence of new mobile platforms heightened the infodemic during the pandemic [47]. Media coverage also affected citizens’ psychological state [48], while information exposure affected citizens’ trust in governments, especially their experiences of lockdown measures [49]. During the initial stage of the pandemic, official social media accounts played an important role in disseminating authoritative information about COVID-19, which resulted in a reduction in uncertainty. Based on this, we propose the following hypothesis:

**Hypothesis** **1** **(H1).** *The information quality of official social media accounts has a significant negative effect on the infodemic*.

### 2.3. Information Cascades and the Infodemic

As rumors and false information started to spread on social media, citizens’ imitation behaviors began to influence information diffusion. Similarly, it triggered uncertainty and fluctuation [50], resulting in information cascades. Individuals with limited official information become reliant on the collective opinions of others as a reference to making their own decisions [19]. Information dissemination, therefore, quickly becomes a dynamic process in which one group imposes their ideas on another group and maintains them, stereotyping the negative characteristics of the group and thus covering up the other characteristics. When negative messages, conveyed by earlier rejection, begin to DOWN cascade, a person may become stigmatized [51].

Disturbances experienced during the initial stage of anxiety-related information processing may lead to subsequent cascades of processing biases [52]. Peer rejection and information processing problems may also have interactive influence, which can lead to intentions to spread rumors [53]. Among investors with a public profile, information cascades increase the offer appeal to early-stage investors who, in turn, attract later-stage investors [54]. Internet users usually imitate other users’ behaviors online, regardless of their own messages [55], which is what happened during the initial stage of the pandemic. Based on this, we propose the following hypotheses:

**Hypothesis** **2** **(H2).** *Information cascades have a significant positive effect on the infodemic*.

**Hypothesis** **3** **(H3).** *Information cascades have a mediation effect on the relationship between IQ and the infodemic*.

### 2.4. Social Support and the Infodemic

The level of social support experienced by citizens affects their mental health far more than the actual structure of personal networks [56]. Social support refers to the feeling of being valued and cared for by a network [57], and is described as the support an individual receives through social connections with other individuals, groups, and the larger community [58], which, in turn, reduces anxiety and panic [59]. Adolescents suffering from severe mental health problems often experience low to medium levels of social support [60]. During the COVID-19 pandemic, citizens who self-isolated experienced significantly higher rates of loneliness and depression than those who did not, with some studies finding that social support is significantly associated with poorer sleep quality and an increased risk of depression [61]. During the outbreak of COVID-19, citizens experienced severe lockdown measures, which limited their social contact with others. As a result, the rates of loneliness, stress, worry, and anxiety grew rapidly [62], which necessitated increased social support. This resulted in some citizens sharing fake news online for different reasons; for example, to seek social support to reduce anxiety [63]. Based on this, we propose the following hypotheses:

**Hypothesis** **4** **(H4).** *Social support has a significant negative effect on the infodemic.*

**Hypothesis** **5** **(H5).** *Social support has a mediation effect on the relationship between IQ and the infodemic.*

### 2.5. Mediation and Moderation Variables and the Infodemic

Social media use, low e-health literacy, and rapid publishing processes are cited as major contributors to the COVID-19 infodemic [1]. Citizens that frequently use social media can experience information overload, which has a significant effect on their mental health [18,64]. Excessive use of social media to seek COVID-19 information may also lead to depression and anxiety [59]. Some people experience difficulties in finding and evaluating information [36], which becomes more serious during public health crises. Ultimately, the COVID-19 infodemic highlighted the poor health literacy of global citizens, which is defined as the cognitive ability of people. During the pandemic, health literacy was perceived as important for preventing COVID-19 with governments investing heavily in education and improved communication [65]. The perceived threat of COVID-19, lower levels of digital health literacy, and rejection of official government social media led to higher levels of COVID-19 misinformation [66]. In this regard, it is understood that health literacy and private social media use may indirectly affect the infodemic. Based on this, we propose the following hypotheses:

**Hypothesis** **6** **(H6).** *Private social media use moderates the relationship between IQ and the infodemic*.

**Hypothesis** **7** **(H7).** *Health literacy moderates the relationship between IQ and the infodemic*.

**Figure 1:**
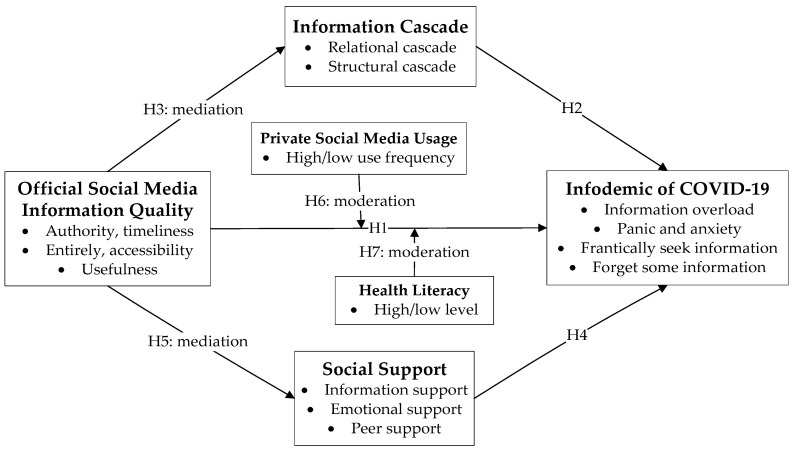
Theoretical Model.

Based on these hypotheses, this study aims to examine the effect of official social media accounts on the infodemic during the first wave of COVID-19 in China. Figure 1 presents our theoretical model. Specifically, we aim to understand how information cascades and social support mediate the relationship between official social media accounts and the infodemic. Moreover, how do health literacy and private social media use moderate the relationship between official social media accounts and the infodemic?

## 3. Methods

### 3.1. Questionnaire and Samples

A questionnaire was written in Chinese, and composed of three sections. The first section explained that the survey was to be completed anonymously and that the data collected would be used for scientific research purposes only. The second section collected participants’ perceptions about the variables amended from many references, including information quality, social support, information cascades, and the infodemic. The measurement items are shown in the *Measures* section, and the modified items of this section were reviewed by a panel of experts, including a Professor who studies government social media, one public health expert, and one data analyst. The third section collected socio-demographic information about participants, their social media use frequency, and their health literacy level, such as gender, age, education level, and household income, as shown in Table 1.

As most citizens were isolated at home during the COVID-19 pandemic, survey invitations were sent electronically with responses being solicited online. The survey was carried out from March to April 2020, with 4152 citizens over the age of 18 years old being randomly invited, including those who have different levels of education and income, as shown in Table 1. Responses were collected anonymously using WeChat and Tencent QQ, both leading Chinese social media platforms. A random sampling strategy, focused on recruiting residents in the COVID-19 outbreak regions of Mainland China, was used. In the beginning, a pilot study was conducted to test the reliability and validity of the constructs. The Cronbach’s α values and KMO values showed good reliability and validity in the preliminary study (0.883 and 0.847, respectively). Then, we sent our questionnaire to all citizens invited; 1515 citizens completed the questionnaire; however, 117 were considered invalid responses. In total, 1398 valid responses were received that covered all provinces that experienced the first wave of the COVID-19 outbreak.

### 3.2. Measures

This study investigated the effects of the information quality of posts published by official social media accounts, information cascades, and the social support on the infodemic during the first wave of the COVID-19 pandemic in China. All scale items were measured using a 5-point Likert-type scale, where 1 = strongly disagree and 5 = strongly agree.

#### 3.2.1. Information Quality (IQ) of Official Social Media Content

Information quality is the degree to which information satisfies users based on their perception [67]. In the context of COVID-19, the information quality of COVID-19-related content was assessed based on its usability and reliability, etc. [68]. In this study, we propose an Information Quality Evaluation Index for Official Social Media (IQEI-OSM) based on user subject cognition, including information expression level, information content, and information utility level. The information quality of COVID-19-related content on social media should be comprehensive (i.e., not omitting important information) and authoritative [69], up-to-date (timeliness) [70], and easy to access and read (accessibility) [65]. The measurement of IQ was completed using a 5-point Likert scale, where the authors asked participants about their perceptions toward the information quality of COVID-19 content posted by official social media accounts. Five statements were used to measure participants’ perceptions, including their agreement toward the following characteristics: (1) authoritative; (2) timeliness; (3) comprehensive; (4) accessibility; and (5) usefulness [71,72,73,74]. The IQEI-OSM had a high internal consistency, as shown in Table 2; the higher scores indicate higher quality of information posted by official social media.

#### 3.2.2. Social Support

Social support is defined as the support accessible to individuals through their social ties with other individuals, groups, and the wider community [58], which affects their preventive health behavior and mental health [57]. The Multi-dimensional Scale of Perceived Social Support (MSPSS), proposed by Zimet et al. [61], is a 12-item measure of perceived adequacy of social support from three sources, including friends, family, and a significant other. Based on prior research, this study measures social support as a multidimensional concept, including information support, emotional support, and peer support, which citizens receive when obtaining COVID-19-related health information from official social media accounts. Seven items were included to measure social support [75,76], including: (1) I would rather visit official social media accounts for COVID-19-related information than ask someone in-person (prefer official); (2) on official social media accounts, I have obtained information about preventing COVID-19 that I never knew from anywhere else (study knowledge); (3) I used the official social media account to deal with stress caused by the COVID-19 pandemic (manage press); (4) while visiting official social media accounts, I felt I have fewer concerns (reduce worry); (5) the health information posted on official social media accounts alleviates my feeling of loneliness (alleviate loneliness); (6) I used official social media accounts to understand other’s experience during the initial stage of COVID-19 (read experience); and (7) I shared the practical advice and suggestions about preventing COVID-19 found on official social media accounts with my friends and family (share advice). The social support index had a high internal consistency, as shown in Table 2, where the higher scores indicate more social support.

#### 3.2.3. Information Cascades

Information cascades occur when individuals observe and act on the behavior of others, regardless of their own information. As a result, they follow the behavior of the preceding individual to reach an optimal state. In this scenario, cascades might cause individuals to make wrong decisions [77]. Because of the zero-sum nature of attention; the amount of information found on private social media accounts draws users’ attention away from official social media accounts. Some information is prevalent while the rest is ignored, which is known as the typical and common ‘long-tail’ phenomenon on social media [78]. Information cascades are measured in the form of relational cascades and structural cascades [50,79]. Four items were used to measure information cascades: (1) I relied on the opinions of others to process information related to COVID-19 (relation cascades1); (2) I relied on the opinions of others to make preventative decisions about COVID-19 (relation cascades2); (3) I relied on social norms to process information about COVID-19 (structural cascades1); and (4) I relied on social norms to make preventative decisions about COVID-19 (structural cascades2). The information cascades index had a high internal consistency, as shown in Table 2, where the higher scores indicate higher levels of information cascades.

#### 3.2.4. The COVID-19 Infodemic

The vicious circle of psychological problems and the spread of rumors were the main features of the COVID-19 infodemic [1]. The measurement of the infodemic was mainly derived from previous studies. Five items were used to measure the COVID-19 infodemic [34,80], namely: (1) during the COVID-19 pandemic, the information I received exceeded my capacity to cope with it (exceeded); (2) during the COVID-19 pandemic, I felt panicky when I saw the amount of information about COVID-19 from different sources (panicky); (3) during the COVID-19 pandemic, I constantly sought information about COVID-19 (excessive seek); (4) because of my excessive information seeking on different media channels, I often forgot to respond to other important messages (forgotten); and (5) during the COVID-19 pandemic, I found it difficult to obtain reliable information when I needed help (difficult). The infodemic index had a high internal consistency, as shown in Table 2, where the higher scores indicate higher levels of the infodemic.

### 3.3. Partial Least Squares Structural Equation Modeling (PLS-SEM)

PLS-SEM is used to estimate complex models with many constructs, indicator variables, and structural paths, without making distributional assumptions about the data, which is useful for exploratory research when examining a developing or less developed theory [81]. Similarly, it can deal with multi-collinearity problems. We used PLS-SEM to examine the effects of the information quality of official social media, information cascades, and social support, on the infodemic using Smart-PLS 3.3.7 software (www.smartpls.com, accessed on 1 April 2022), for two reasons. First, based on the SMCC theory, we added two variables (i.e., information cascades and social support) to explain the underlying mechanisms of the relationship between official social media accounts and the infodemic, which can be seen as a less developed theory. Second, PLS-SEM can report the R^2^ values of each endogenous latent variable.

## 4. Results

### 4.1. The Measurement Model

**Figure 2:**
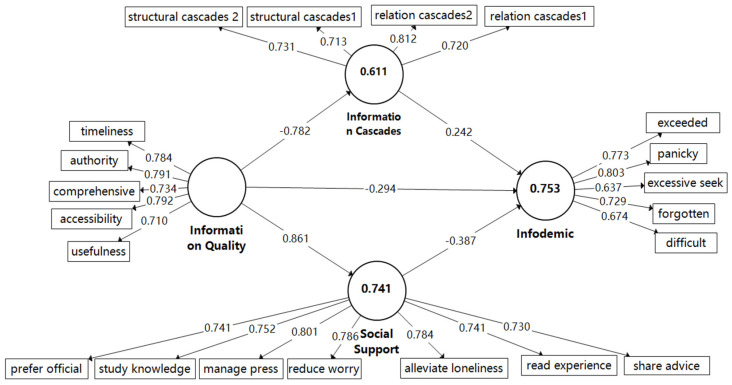
PLS-SEM analysis results. Note: All the numbers in this figure are standardized.

This study conducted the internal consistency test using Smart-PLS 3.3.7 statistical software. Composite Reliability (CR) values were calculated to test the reliability and internal consistency of the scale. The Cronbach’s α is another measurement of internal consistency reliability, which is a less precise measure than CR. Rho_A lies between Cronbach’s α and CR, which may represent a good compromise [82]. Average Variance Extracted (AVE) is used to assess the convergent validity of each construct’s measure. The Cranach’s α of each subscale was >0.6, which indicates that the survey data were highly reliable. The outer loadings ranged from 0.637 to 0.812, as shown in Figure 2, which exceeds the minimum value of 0.60. The Cronbach’s α values ranged from 0.732 to 0.880, which showed a satisfactory internal consistency level. The CR values ranged from 0.833 to 0.907, with ‘satisfactory to good’, indicating the instrument had good internal consistency. The AVE values were higher than 0.50, which indicates that the four constructs explain more than 50% of the variance of their own items [83]. Further, the range of the Variance Inflation Factor (VIF) was 1.280 to 2.068, as shown in Table 2, and all were less than 5, which shows that there was no significant multi-collinearity risk.

Discriminant validity was completed using Smart-PLS by the criterion provided by Fronell–Larcker [84]. Table 3 shows that the minimum square root values of the AVE along the diagonal line were higher than the correlation values between latent constructs in each column, which meets the above criterion and indicates that the measurement model had an acceptable discriminant validity level.

### 4.2. The Structural Model

#### 4.2.1. Standardized Path Coefficient

Results show that PLS-SEM had an adequate fit since the model’s fit indicates that d_ULS (=0.592) and d_G (=0.223) were less than 0.95, SRME (=0.051) was less than 0.08, and NFI index was 0.893. The R^2^ values of the Infodemic, Information Cascades, and Social Support were 0.753, 0.611, and 0.741, respectively, indicating that the model was moderate and had a substantial fit [85].

Our results show that the information quality of content posted by official social media accounts (β = −0.294, *p* < 0.001) had a significant negative effect on the infodemic, while information cascades (β = 0.242, *p* < 0.001) had a significant positive effect on the infodemic. Social Support (β = −0.387, *p* < 0.001) had a significant negative effect on the infodemic. Thus, hypotheses H1, H2, and H4 are supported. Approximately 75.3% of the variance in the COVID-19 infodemic (see Figure 2) was driven by the significant influence of information quality, information cascades, and social support. The path coefficients, t-statistics, and *p*-values, of the hypotheses, are shown in Figure 2 and Table 4, respectively.

All path coefficients are standardized enabling us to compare their absolute value. The absolute value of social support (0.387) is higher than information quality (0.294) and information cascades (0.242), indicating that social support has the greatest negative direct effect on the infodemic. This means that the higher level of social support received, the lower level of the infodemic. The absolute value of information quality is lower than social support, indicating a greater negative direct effect on the infodemic, which means that the higher level of information quality, the lower level of the infodemic. However, it should be noted that information cascades have a positive effect on the infodemic, which means that the higher the level of information cascades, the higher the level of the infodemic.

#### 4.2.2. Mediation Analysis

The two models have two mediation effects, namely: Information quality → Social Support → Infodemic, and Information quality → Information cascades → Infodemic. To examine the significance of indirect effects, the bootstrapping method was used with either data normality distribution or not [86]. Since the results show that official social media accounts were the key factor in controlling the infodemic, the underlying mechanisms of this factor are further explored through meditation analysis using Smart-PLS and Bootstrapping with 5000 subsamples. The bootstrap method was used to examine the hypothesized relationships and sampling distribution as a measure of accuracy using random sampling methods to ensure consistency in results [81]. Mediation analysis was conducted to better understand the relationship between the information quality of official social media accounts and the infodemic, and the mediation effects of information cascades and social support were shown with 95% Confidence Intervals (CI).

Our results revealed that with 95% CI, if information cascades were taken as the mediator variable, the mediation effect was significant (β = −0.189, Boot CI (−0.227, −0.151)). If social support was taken as the mediator variable, the mediation effect was significant (β = −0.333, Boot CI (−0.388, −0.280)). In general, the information quality of content posted by official social media accounts has both a direct and indirect effect on the infodemic, and mediation through both information cascades and social support. This indicates that official social media accounts contained the COVID-19 infodemic and, therefore, hypotheses H3 and H5 are supported.

### 4.3. Moderating Analysis

**Figure 3:**
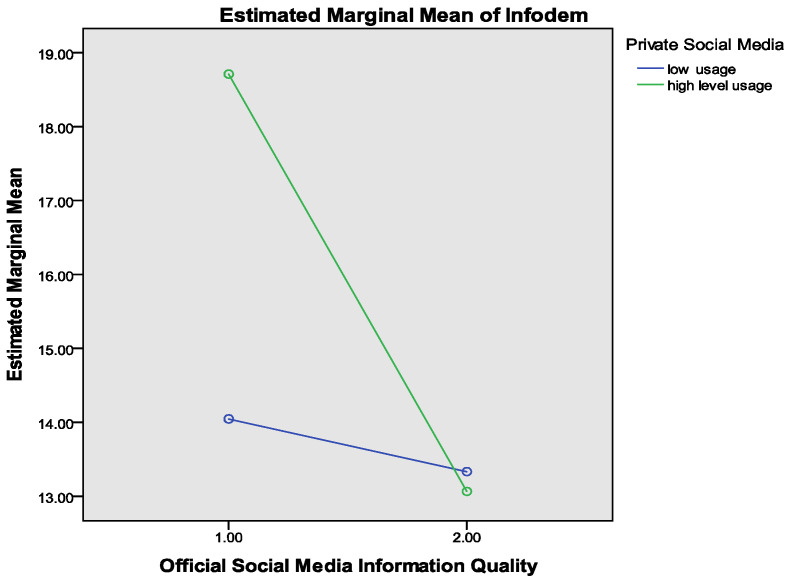
Moderation analysis for the effect of private social media use on the infodemic.

**Figure 4:**
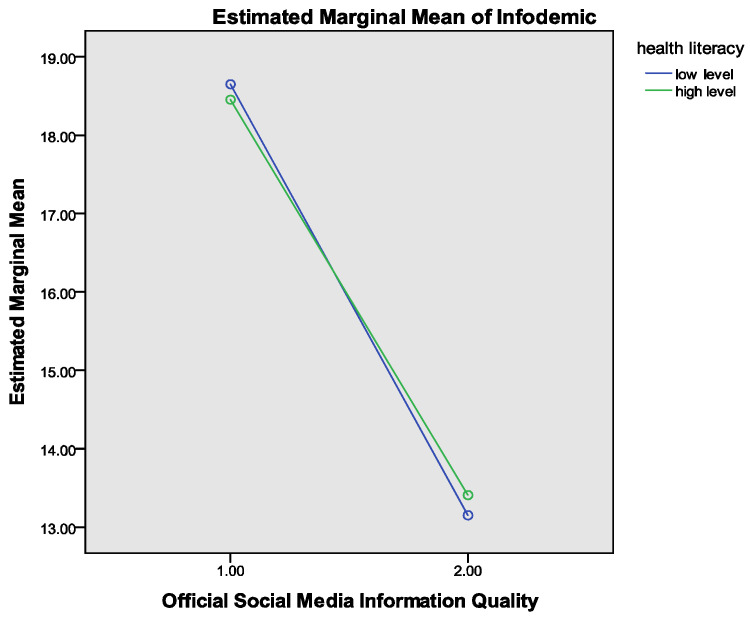
Moderation analysis for the effect of health literacy on the infodemic.

The two-factor analysis of ANOVA with interaction was used to conduct a moderation/interaction effect analysis with visualization [87]. The results of the moderation effect are shown in Figure 3 and Figure 4. To further examine the effect of information posted by official social media accounts on the infodemic under different conditions, this study coded the official social media information quality into two groups (1 = low level, 2 = high level). Similarly, private social media use was coded into two groups (0 = low usage, 1 = high usage), as shown in Figure 3. Then, a 2 (two groups of IQ) × 2 (two groups of private social media use) off our groups ANOVA was performed, taking different levels of information quality perceptions and private social media use as independent variables, and the infodemic as the dependent variable. The main effect of both information quality (F = 142.347, *p* < 0.001) and private social media use (F = 68.177, *p* < 0.001) were significant. The interaction effect of information quality and private social media use was also significant (F = 85.637, *p* < 0.001). The line of high usage and the line of low usage of private social media intersected, and the slope of high usage was greater than the low usage (see Figure 3). This finding indicates that private social media usage positively moderated the relationship between information quality and the infodemic.

At the same time, health literacy was coded into two groups (0 = low level, 1 = high level), as shown in Figure 4. Then, a 2 (two groups of IQ) × 2 (two groups of health literacy) (see Figure 4) of four groups ANOVA was performed, taking different levels of information quality perceptions and health literacy as independent variables, and the infodemic as the dependent variable. The main effect of information quality (F = 1182.015, *p* < 0.001) was significant. However, the main effect of health literacy was not significant (F = 0.040, *p* > 0.05). It is well-known that the significance of moderating effects cannot be judged by simply relying on the significance of the product terms. It should be judged comprehensively by whether they have interaction points in the interaction graph or not. Therefore, the interaction graphs were drawn, as shown in Figure 4, with results revealing that the line of two levels of health literacy had an intersection while the slope of the high level is smaller than the low level. This indicates that health literacy negatively moderated the relationship between information quality and the infodemic and, therefore, hypotheses H6 and H7 are supported.

### 4.4. Predict Partial Least Squares (PLS) Model

The PLS predict algorithm uses training and hold out samples to generate and evaluate predictions from PLS path model estimations, which means it combines aspects of out-of-sample prediction and in-sample explanatory power [88]. The PLS predict results are shown in Table 5. The Q^2^_predict statistic values of PLS-SEM outperform most LM benchmarks [89]. Meanwhile, only in addition to the indicator of read experience, the other indicators in the PLS-SEM analysis have lower RMSE and MAE values compared to the LM benchmark [85], which indicates that the structural model has higher explanatory power and predictive power.

## 5. Discussion

This study provides valuable insights into the effects of official social media accounts on the infodemic during the initial stage of COVID-19. Recent studies have focused their efforts on examining the effects of the infodemic on citizens’ psychological issues and mental health [6,34], and how private social media use has affected the infodemic [1,12,45]. For example, some studies have found that commercial media positively affects psychological anxiety, but that official government media has no effect on psychological anxiety [90]. However, the underlying mechanisms of how official social media accounts affect the infodemic have received little attention. Specifically, during the pandemic, it has not previously been understood how official social media accounts affect the infodemic by the mediation effects of information cascades and social support, and the moderation effects of private social media use and health literacy.

Our results show that the information quality of content posted by official social media accounts and the social support provided have a significant negative effect on the infodemic. Information cascades have a significant positive effect on the infodemic. Mediation analyses were conducted to explore the underlying mechanisms of the relationship between IQ and the infodemic with results revealing that both information cascades and social support mediate the relationship between IQ and the infodemic. In addition, moderation analyses were completed to explore the underlying mechanisms of the relationship between IQ and the infodemic, with results indicating that private social media use and health literacy moderate the relationship between IQ and the infodemic.

These findings demonstrate the underlying mechanisms of the relationship between official social media accounts and the infodemic. In the context of public health crises, citizens tend to seek information to alleviate uncertainty (e.g., public health, personal and family safety, and recovery efforts) [91]. Our findings show that official social media accounts have controlled the infodemic and have increased the social support provided to citizens. In other words, it has alleviated citizens’ uncertainty regarding COVID-19. It is noted, however, that it is necessary to guide citizens in using and promoting the use of official public health organizations’ websites and official social media accounts when seeking health information related to COVID-19 [92]. On the other hand, the rational use of official social media should be promoted to prevent the dissemination of misinformation. Similarly, social media users should be trained to identify misinformation by using official information sources only and scientific digital health literacy cultivation [36].

Information cascades and social support were found to be important mediation variables in explaining how official social media accounts affected the infodemic during the first wave of COVID-19. When citizens were exposed to excessive information related to COVID-19, they tended to choose information that was useful to themselves. Meanwhile, they often sought social support (e.g., information support and emotional support) to alleviate the uncertainty experienced. If citizens made decisions by relying on the opinions of others or social norms, it indicates that information cascades have occurred. During the first wave of COVID-19, commercial media circulated an overload of information, with epidemic information being pushed to users continuously [90], with reliable and authoritative information being important for designing and conducting preventive measures to raise health-protective awareness [14]. This study shows that official social media can provide high-quality epidemic information to citizens, which can increase social support and reduce information cascades. This study also confirmed that the greater use of social media can lead to more social support [19].

Different information sources were shown to have different effects on the infodemic [90]. Private social media use is found to be a double-edged sword, which was found to be a major source of rumors or misinformation during emergencies in prior studies; on the other hand, it also plays a key role in communicating health information [93]. This finding shows that private social media use positively moderates the relationship between IQ and the infodemic, indicating that excessive use of private social media increases public anxiety and leads to an infodemic [19]. Therefore, this finding proves that citizens should take a break from private social media, and rationality use both official social media and private social media during public health crises. Health literacy was also found to negatively moderate the relationship between IQ and the infodemic. When citizens are faced with uncertainty, they are in a state of anxiety and depression, and their health literacy provides little help in identifying valid health information.

### 5.1. Theoretical and Practical Implications

This study constructed a theoretical model to uncover the underlying mechanisms of the relationship between official social media accounts and the infodemic during the first wave of COVID-19 in Mainland China. The proposed model made several contributions to previous studies by integrating two further variables (i.e., information cascades and social support) to investigate the infodemic problem ongoing with the COVID-19 pandemic. We treated official social media accounts as a cue to controlling the infodemic, while information cascades and social support were used as the mediation variables, which, as we know, have not been proven in prior research. Similarly, we considered the moderation effects of private social media use and health literacy. Thus, this paper calls for more research into the underlying mechanisms of the determinants of the infodemic.

Policy implications can also be derived from this study to develop strategies for controlling future infodemics during public health crises. The outbreak of COVID-19 was accompanied by the mass dissemination of unvalidated information by private social media accounts. If authoritative information about COVID-19 was not published in a timely fashion, it may have caused the further dissemination of false information. This study provides evidence-based implications to control the COVID-19 infodemic. First, the public agencies that manage official social media accounts should improve the usefulness, timeliness, availability, and authoritative nature of the information provided through enhancing the professionalism of practitioners. Public agencies should also establish a release system to control information quality, and use social media and the Internet rationally to encourage citizens to interact with public agencies [94], and to pay greater attention to credible and authoritative sources and fact-checkers about the COVID-19 pandemic [95].

Secondly, official social media accounts should set an example for private social media users and commercial media accounts, such as forging a user-centered, fact-based, and collaborative response to the pandemic. Official social media accounts not only alleviate citizens’ anxiety and uncertainty through the dissemination of authoritative reports, but also carry out useful preventive measures and touching epidemic stories to increase social support, inspiring citizens to fight against COVID-19 together. In special, official social media accounts should establish information communication mechanisms for sharing pandemic information resources between official social media accounts and big individual private social media accounts. Therefore, official social media accounts can help manage citizens’ stress and health risks and should, therefore, convey information to citizens with empathy, scientific and rational evidence, and personal experience, and encourage them to share the content with friends, family, and peers, etc., so as to increase the social support and reduce potential information cascades.

Thirdly, when faced with large amounts of media and health-related information, local governments should formulate rules to regulate the dissemination of pandemic information by private social media accounts. Health literacy may also help citizens better understand the reasons behind governments’ and public health agencies’ preventive recommendations and make protective and preventive actions quickly. In the later stages of COVID-19, citizens showed a good level of knowledge about the disease [96] but, in the initial stages, citizens almost knew nothing about it. Hence, local governments and public health agencies should popularize common health knowledge, while citizens should enhance their health literacy to enable larger-scale psychological prevention of fake news [97]. Meanwhile, local governments should encourage citizens to take social responsibility and encourage people to take the initiative in pandemic prevention.

### 5.2. Limitations and Future Studies

This study has several limitations. First, the survey was administered during the first wave of the COVID-19 pandemic in Mainland China, which relied on respondents’ self-reporting data online. It lacked in-depth interviews with participants, which could help deepen cognition and the importance of official social media accounts and how they helped control the infodemic during the pandemic. Second, in most cases, information cascades were calculated using big data, with the study measuring it using four items. Future research may mine public comments from official social media accounts, and calculate their forwarding, interaction, and liking behaviors. Similarly, studies can analyze their emotional tendency to estimate what support is provided to citizens and uncover how official social media accounts affect citizens’ emotions. Third, only one round of the online survey was conducted. Fourth, some respondents might have been relatively calm and objective to participate in the survey, but some might have felt very anxious and uncomfortable when completing the questionnaire. Fifth, we only examined the effect of information quality of official social media accounts on the infodemic; future study will try to examine the impact of official social media accounts’ response strategies on the infodemic. Finally, there have been several subsequent waves of COVID-19 outbreaks in China since the survey was conducted and, therefore, longitudinal and comparative studies can be conducted in the future.

## 6. Conclusions

This study provided empirical evidence on the effects of official social media accounts on the COVID-19 infodemic and gave insights for uncovering the underlying mechanisms of the infodemic by analyzing the essential roles of the information quality of official social media accounts, the mediation effects of information cascades and social support, and the moderation effects of private social media use and health literacy. Our findings provide policy implications for controlling future infodemics and can help public health agencies that manage official social media accounts improve their information quality, increase social support, and decrease information cascades.

## Figures and Tables

**Table 1:** Background information.

Variable	Category	Number	Percentage (%)
Gender	Male	685	49.0%
	Female	713	51.0%
Age	18–30 years old	323	23.1%
	31–40 years old	504	36.1%
	41–50 years old	375	26.8%
	51–60 years old	128	9.2%
	More than 60 years old	68	4.9%
Education	Junior school or below	120	8.5%
	Senior high school	176	12.6%
	Associate degree	564	40.3%
	Bachelor degree	433	31.0%
	Master’s degree or Ph.D.	105	7.5%
Annual Household Income (Chinese yuan)	Less than 30,000	37	2.6%
	30,000–100,000	825	59%
	110,000–200,000	388	27.8%
	More than 200,000	148	10.6%

**Table 2:** Construct Reliability and Validity.

	Cronbach’s α	Rho_A	CR	AVE	VIF Range
Infodemic	0.773	0.786	0.847	0.527	1.28–01.665
Information Cascades	0.732	0.740	0.833	0.555	1.328–1.540
Information Quality	0.820	0.824	0.874	0.582	1.458–1.716
Support	0.880	0.881	0.907	0.582	1.650–2.068

Note: VIF range, VIF range of each item.

**Table 3:** Discriminant Validity.

	Quality	Cascades	Support	Infodemic
Information Quality	0.763			
Information Cascades	−0.590 **	0.745		
Support	0.660 **	−0.612 **	0.763	
Infodemic	−0.558 **	0.573 **	−0.555 **	0.726

Number of sample = 1398; the diagonal line is the square root value of AVE, while the other values are the correlation coefficients between variables; * <0.05; ** <0.01; *** <0.001.

**Table 4:** Bootstrapping analysis.

Path	O.S.	Sample	S.D.	t	*p*
Cascades → Infodemic	0.242	0.243	0.029	8.366	0.000
Quality → Infodemic	−0.294	−0.293	0.036	8.111	0.000
Quality → Cascades	−0.782	−0.782	0.012	63.292	0.000
Quality → Support	0.861	0.861	0.008	107.155	0.000
Support → Infodemic	−0.387	−0.388	0.036	10.664	0.000

Note: O.S., Original Sample; S.D., Standard Deviation; t, T Statistics.

**Table 5:** PLS predict results.

	PLS-SEM			LM Benchmark		
	RMSE	MAE	Q^2^_Predict	RMSE	MAE	Q^2^_Predict
overstretched	1.018	0.793	0.401	1.013	0.787	0.408
forgotten	1.009	0.839	0.351	1.010	0.839	0.349
refresh	1.000	0.851	0.245	1.001	0.852	0.242
anxiety	0.935	0.745	0.466	0.934	0.743	0.466
difficult	1.011	0.790	0.282	1.014	0.795	0.277
Relation cascades1	1.063	0.898	0.296	1.066	0.900	0.292
Structural cascades2	1.019	0.831	0.317	1.020	0.830	0.315
Structural cascades1	1.047	0.873	0.307	1.050	0.874	0.302
Relation cascades2	0.943	0.747	0.432	0.946	0.749	0.428
Study knowledge	0.955	0.723	0.391	0.958	0.724	0.388
Alleviate loneliness	0.915	0.721	0.450	0.918	0.722	0.446
Reduce worry	0.952	0.742	0.461	0.953	0.740	0.460
Prefer official	0.929	0.715	0.418	0.930	0.717	0.416
Share advice	0.949	0.735	0.386	0.952	0.738	0.382
Manage press	0.915	0.723	0.474	0.915	0.723	0.474
Read experience	0.917	0.713	0.427	0.915	0.709	0.430

## Data Availability

The raw data presented in this paper are available from the authors, without undue reservation.
